# 2058 miRNA manipulation to improve CFTR correction in cystic fibrosis

**DOI:** 10.1017/cts.2018.96

**Published:** 2018-11-21

**Authors:** William Thomas Harris, Farruk Kabir

**Affiliations:** University of Alabama at Birmingham

## Abstract

OBJECTIVES/SPECIFIC AIMS: CFTR is the mutant protein that causes cystic fibrosis (CF), a fatal respiratory diseases affecting 1 in 3500 children. CFTR modulators are small molecules that directly address mutant CFTR function. Improving correction of the F508del CFTR mutation (affecting 90% of CF patients) is one of the most pressing unmet needs in CF. Currently available F508del therapeutics only marginally improve CF, In vitro, we have identified a miRNA that impairs utility of CFTR directed therapies. miR-145 is upregulated by TGF-β (a genetic modifier of CF lung disease) with a direct binding site on the 3’-untranslated region of CFTR mRNA. Binding of miR-145 to CFTR destabilizes mRNA transcript and impedes protein translation. Overexpression of miR-145 abolishes benefit of F508del CFTR correction. Antagonists to miR-145 block TGF-β suppression of CFTR function and augment response to CFTR correction. This project evaluate in vivo impact of TGF-beta and miRNA manipulation on CFTR functional readouts including nasal potential difference (NPD) and short circuit current (Isc) across tracheal explants in addition to standard biochemical measures. METHODS/STUDY POPULATION: Wild-type Sprague-Dawley rats were inoculated with an adenoviral vector containing bioactive TGF-beta or sham at 1×10^9^ pfu/animal placed in the left nares. Seven days post-inoculation, functional, and biochemical measures were conducted. NPD was measured with a microelectrode placed in the left nare and grounded the tail. The nare was sequentially perfused with standard Ringer’s solution, amiloride (to block the ENaC sodium channel), low chloride Ringer’s (to stimulate chloride efflux), forskolin (to open the CFTR channel) and CFTRinh-172 (to block the CFTR channel. Tracheal explants were harvested, microdissected, and placed on modified Ussing chambers. RESULTS/ANTICIPATED RESULTS: We have inoculated WT rats with bioactive TGF-β Versus sham delivered by intranasal inoculation of an adenoviral vector. Functional readout of CFTR function is by Isc across tracheal epithelia and NPD. Lung homogenates are analyzed for TGF-β signaling, miRNA expression, and CFTR transcripts. Both tracheal explants and NPD indicate TGF-β stimulation diminishes CFTR function in vivo. In tracheal explants, TGF-β exposure diminishes CFTR response to forskolin-stimulation by 75%. Loss of current after CFTR inhibition (CFTRinh-172) is halved. By nasal PD, TGF-β inoculation similarly halves the bioelectric response to low chloride and forskolin stimulation. Evaluation by qPCR reveals a strong increase in TGF-β signaling demarcated by PAI-1, prompting a reduction in CFTR mRNA. miR-145 is expressed highly in rat pulmonary tissue, but no change in overall miR-145 levels was detected between TGF-β and sham exposed rats. This finding reflects what we have observed in human lungs, with a localized increased miR-145 expression in CF epithelia, but similarly high levels of miR-145 in both CF and non-CF whole lung homogenates. Although expressed at lower levels than miR-145, we did find increased expression in TGF-β relevant miR-101, miR-494, and miR-144 that have a predicted binding site on rat 3’-UTR in TGF-β exposed Versus sham lungs. DISCUSSION/SIGNIFICANCE OF IMPACT: Our data indicate the relevance of TGF-β stimulation to suppress CFTR synthesis and function in vivo. Future work will evaluate whether these additional miRNA with CFTR binding sites may mediate TGF-β suppression of CFTR in the rat model, and the utility of miRNA manipulation to augment F508del CFTR correction.

